# Recent Developments in Penem Antibiotics: Structural and Therapeutic Perspectives

**DOI:** 10.3390/molecules30102126

**Published:** 2025-05-11

**Authors:** Aura Rusu, Octavia-Laura Oancea, Elena Donici, Livia Uncu

**Affiliations:** 1 Pharmaceutical and Therapeutic Chemistry Department, Faculty of Pharmacy, George Emil Palade University of Medicine, Pharmacy, Science and Technology of Targu Mures, 540142 Targu Mures, Romania; aura.rusu@umfst.ro; 2 Organic Chemistry Department, Faculty of Pharmacy, George Emil Palade University of Medicine, Pharmacy, Science and Technology of Targu Mures, 540142 Targu Mures, Romania; 3 Scientific Center for Drug Research, Pharmaceutical and Toxicological Chemistry Department, “Nicolae Testemitanu” State University of Medicine and Pharmacy, 165 Bd. Stefan Cel Mare si Sfant, MD-2004 Chisinau, Moldova; elena.donici@usmf.md (E.D.); livia.uncu@usmf.md (L.U.)

**Keywords:** penems, β-lactam antibiotics, sulopenem, faropenem, antibacterial activity, antibacterial resistance, urinary tract infections (UTIs), penem derivatives, β-lactamase inhibitors, carbapenems

## Abstract

This review examines the latest progress of β-lactam antibiotics, focusing on penems. Penems are distinguished by their unique structural characteristics and remarkable antibacterial activity. The structural characteristics of the class that differentiate it from carbapenems or monobactams are addressed. Notable representatives such as sulopenem and faropenem are discussed. Faropenem’s stability and efficacy against resistant bacterial strains emphasize the therapeutic potential of penems. The review highlights sulopenem’s recent FDA approval, marking a key point in treating uncomplicated urinary tract infections caused by specific bacteria in adult women with minimal or no other options for oral antibiotic treatment. The review covers sulopenem’s structural considerations, physicochemical properties, mechanisms of action, antibacterial activity, and clinical pharmacology. The development of penem derivatives is also addressed, emphasizing their potential in combating resistant bacterial infections. Despite having few approved representatives, penems show promising prospects for future design and may significantly contribute to the fight against bacterial resistance. The review also highlights the challenges and future possibilities in penem research, including the need for improved oral bioavailability and the potential for combination therapies with β-lactamase inhibitors. Overall, penems are valuable antibacterial agents in the antimicrobial arsenal, offering hope in the ongoing battle against multidrug-resistant pathogens.

## 1. Introduction

**Figure 1:**
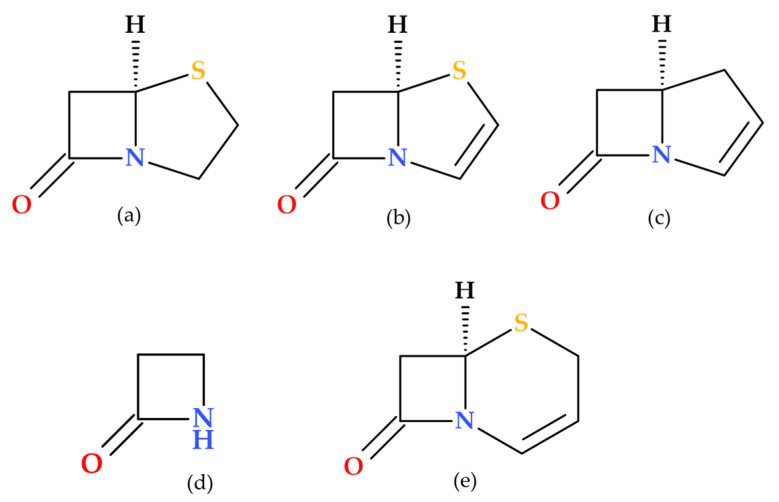
General structures of the essential bicycle cores of β-lactam antibiotics classes: (**a**) penam, (**b**) penem, (**c**) carbapenem, (**d**) azetidin-2-one (monobactam), and (**e**) cephem [1,5].

β-lactam antibiotics are among the most commonly prescribed antibiotics for treating bacterial infections. Penicillin was the first antibiotic discovered by Alexander Fleming in 1928; its molecule contains a distinctive β-lactam ring (azetidine-2-one), which confers its antibacterial properties. The carbonyl of the β-lactam ring is very reactive, exhibiting a stronger electrophilic character than the carbonyl of the exocyclic amide (at the side chain level of the overall structure) of β-lactam antibiotics [1]. Thus, the main characteristic of β-lactam antibiotics is the β-lactam pharmacophore function fused with another heterocycle (a non-coplanar bicyclic scaffold), which may differ from one subclass to another. The class of β-lactam antibiotics includes several subclasses (penicillins, cephalosporins, carbapenems, penems, and monobactams) that differ in the heterocycle fused to the β-lactam ring. For example, the thiazolidine fused with the β-lactam ring is found in the penam core, specific to penicillins. Similarly, the dihydrothiazine is found in the cephem core, specific to cephalosporins (Figure 1). Additionally, β-lactamase inhibitors such as sulbactam, tazobactam, and clavulanic acid also incorporate a β-lactam ring in their structures [1,2,3,4].

β-lactam antibiotics exert their bactericidal effect by inhibiting bacterial cell wall synthesis. Thus, β-lactam antibiotics target the final stage of biosynthesis of peptidoglycan, a critical component of the bacterial cell wall that provides structural integrity. Specifically, β-lactam antibiotics target transpeptidase enzymes responsible for cross-linking the peptidoglycan cell wall by mimicking the *D*-Ala-*D*-Ala dipeptide substrate of transpeptidase enzymes—also known as penicillin-binding proteins (PBPs)—which catalyze the cross-linking of peptidoglycan strands. The beta-lactam ring forms a covalent bond with the active site serine of PBPs, creating a stable penicilloyl–enzyme complex that irreversibly inhibits the enzyme. The action on PBPs in the cell membrane occurs through the covalent binding and opening of the β-lactam ring. The enzymatic activity of PBPs is essential in the final stages of peptidoglycan synthesis. The inhibition of PBPs prevents the formation of cross-links in the peptidoglycan matrix, weakening the cell wall and leading to osmotic instability, cell lysis, and, ultimately, bacterial death. Consequently, β-lactam antibiotics are classified as bactericidal agents [1,4,6]. The selective toxicity of β-lactam antibiotics arises from the absence of peptidoglycan and PBPs in human cells, making them safe and effective antibacterial agents [1].

β-lactam antibiotics are generally well tolerated, but their use is contraindicated in patients with a history of severe hypersensitivity reactions, including anaphylaxis or severe cutaneous adverse reactions (e.g., Stevens–Johnson syndrome, toxic epidermal necrolysis) [7].

Penems are β-lactam antibiotics that can be synthesized entirely or derived from penams. Penems possess a β-lactam ring and consequently have a mechanism of action similar to the β-lactam antibiotic class [1]. The first penem was synthesized by Woodward R.B. as a combination of the penam and cephem nuclei, creating a hybrid β-lactam structure that does not occur naturally. Penems are fully synthetic and feature a thiazoline ring in place of the pyrroline ring found in carbapenems. The modification alters the ring conformation, reducing the intra-molecular strain and influencing their pharmacokinetic properties [8,9].

Bacterial diseases significantly impact human health alongside the widespread use of β-lactam antibiotics [10]. Bacterial resistance typically arises due to self-medication, genetic variations or mutations within the bacteria, and phenotypic changes, including those caused by β-lactam antibiotics [11]. Despite the effectiveness of the approved β-lactam antibiotics, bacteria have developed resistance mechanisms against all major classes of β-lactam antibiotics. Specifically, bacterial resistance to carbapenems involves several mechanisms—enzymatic degradation by carbapenemases (including class A KPC-2, class B metallo-β-lactamases, and class D oxacillinases), target modification through altered PBPs (e.g., PBP2a in methicillin-resistant *Staphylococcus aureus* (MRSA)), and the regulation of drug entry and efflux. Bacteria can reduce carbapenem entry by modifying or losing porins and expelling the drug using efflux pumps (in *Pseudomonas aeruginosa* and *Acinetobacter baumannii*) [10,12]. Bacterial resistance to the penem class of β-lactam antibiotics (e.g., faropenem and sulopenem) primarily involves enzymatic degradation by β-lactamases, including carbapenemases and metallo-β-lactamases. Additionally, bacteria can modify PBPs to reduce the binding affinity of β-lactam antibiotics and employ efflux pumps to expel the drugs from the cell, decreasing their intracellular concentration. Alterations in outer membrane proteins can also limit the penetration of faropenem and sulopenem into bacterial cells, further contributing to resistance. The combined mechanisms highlight the complexity of bacterial resistance and the challenges in developing effective treatments [13,14,15].

Our review aims to provide a comprehensive overview of recent developments in penem antibiotics, focusing on their structural characteristics, antibacterial activity, and therapeutic potential. The paper discusses notable representatives, such as faropenem, known for its stability and efficacy against resistant bacterial strains and sulopenem. Sulopenem was recently approved by the USA Food and Drug Administration (FDA) for treating uncomplicated urinary tract infections (UTIs) in adult women. It covers sulopenem’s structural considerations, mechanisms of action, antibacterial activity, and clinical pharmacology. The review emphasizes the promising prospects of penems in combating bacterial resistance, highlighting the development of penem derivatives and their potential in treating resistant bacterial infections. Additionally, the paper addresses the challenges and future possibilities of penem research.

## 2. Penems Used in Therapy

Penem antibiotics are not natural compounds, but are obtained through synthesis [1,9,16].

### 2.1. Structural Considerations Regarding Penems

Penems are a distinct subclass of β-lactam antibiotics characterized by their β-lactam ring fused with a thiazoline ring (penem heterocycle). Their unique structure differentiates them from other β-lactam antibiotics, such as penicillins, cephalosporins, and carbapenems. Similar to β-lactam antibiotics, penems inhibit PBPs, which results in bactericidal action [1].

**Figure 2:**
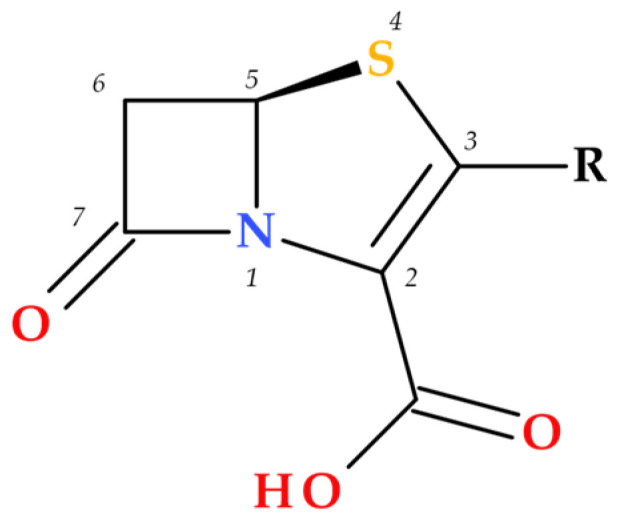
The classification of penems based on the C3 side chain (R): alkylpenems, arylpenems, aminopenems, oxypenems, and thiopenems [17].

Penems can be obtained by total synthesis or from penams. Five subclasses of the penem family can be identified based on the characteristics of the C3 side chain, which determines the molecules’ pharmacokinetic and toxicological characteristics: alkylpenems, arylpenems, aminopenems, oxypenems, and thiopenems (Figure 2). Our review follows the standard numbering convention for penem structures, where the nitrogen atom is designated at position 1 and the sulfur atom at position 4. A C2–C3 double bond distinguishes penems from penams, promoting enamine resonance (a type of electron delocalization), weakening the C7–N1 bond, and increasing β-lactam hydrolysis susceptibility. Consequently, penems are less stable than penams, which limits their chemical reactivity and pharmaceutical applications [17].

**Figure 3:**
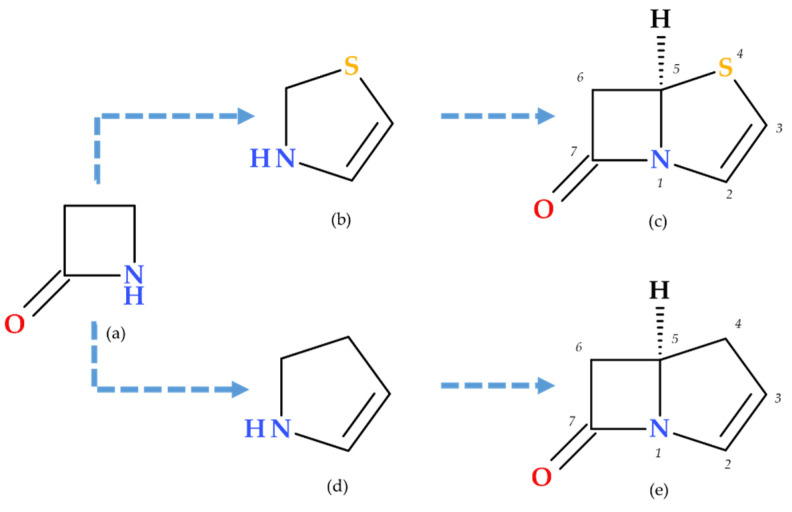
The central core of carbapenems, penems, and their constituent heterocycles: (**a**) azetidin-2-one, (**b**) thiazoline (2,3-dihydrothiazole), (**c**) penem ((5*R*)-4-thia-1-azabicyclo [3.2.0]hept-2-en-7-one), (**d**) pyrroline (2,3-dihydro-1*H*-pyrrole), and (**e**) carbapenem ((5*R*)-1-azabicyclo [3.2.0]hept-2-en-7-one); the penem and carbapenem heterocycles are numbered [1,5].

Due to the fused thiazoline that contains sulfur (2,3-dihydrothiazole), the molecular structure of penems is similar to that of penicillins, except for the double bond C2–C3 on the bicycle (Figure 3). Penem antibiotics are obtained through synthesis, in contrast to the carbapenem class. Structurally, penems represent a bivalent isostere of carbapenem, commonly known as penem, achieved by substituting a sulfur atom for a methylene group in the five-membered ring (pyrroline is replaced by thiazoline) fused to the β-lactam ring [1].

One common characteristic of all penems is the double bond that connects C2 and C3 atoms. The double bond increases the β-lactam ring’s reactivity to nucleophiles, similar to the serine in PBP active sites, and the double bond between C3 and C4 positions in cephalosporins. Thus, the double bond is an essential characteristic of the cephalosporins present in penems’ structure [1,9,18,19].

The β-lactam ring becomes more reactive in two ways when a double bond is conjugated to the β-lactam nitrogen. Initially, the amide nitrogen’s double bond competes for the unshared nitrogen electrons, decreasing their delocalization into the nearby carbonyl group and increasing the β-lactam ring’s ground state energy. In addition, the conjugated double bond lowers the transition state energy required for β-lactam ring cleavage by decreasing the basicity of the departing amine nitrogen, which improves the leaving group. As a result, the β-lactam ring becomes more reactive to various nucleophiles, such as water (hydrolysis), amines, and the serine residues found in PBP’s active sites. Furthermore, when a thiazoline ring with sulfur at position 4 instead of carbon in a pyrroline ring is present, the five-membered ring’s conformation is changed, and intra-ring tension is decreased due to a longer C–S bond length and a lower C–S–C bond angle [9].

**Figure 4:**
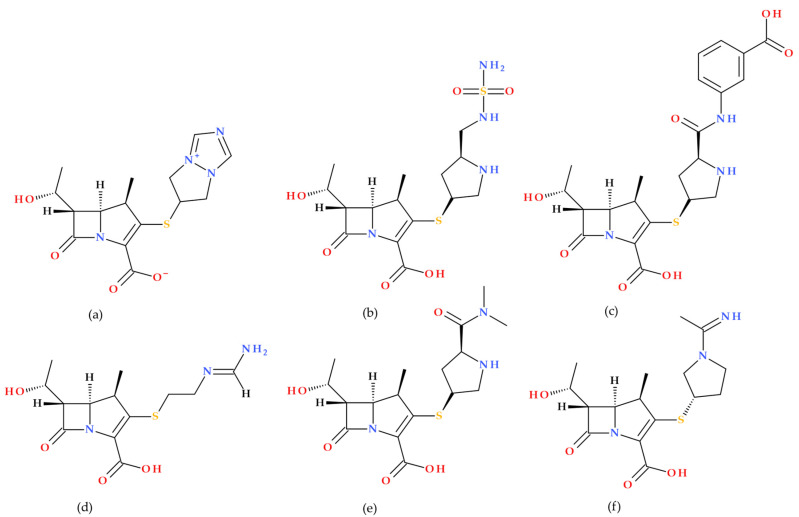
Molecular structure of carbapenems used in therapy (approved by the FDA, European Medicines Agency (EMA) or in the countries of origin): (**a**) Biapenem; (**b**) Doripenem; (**c**) Ertapenem; (**d**) Imipenem; (**e**) Meropenem; (**f**) Panipenem [5,20,21]; Doripenem was withdrawn in the European Union (EU) [22].

The substituents at positions C3 and C4 represent the key difference between penem and carbapenem antibiotics (Figure 4) [1].

### 2.2. Faropenem

**Figure 5:**
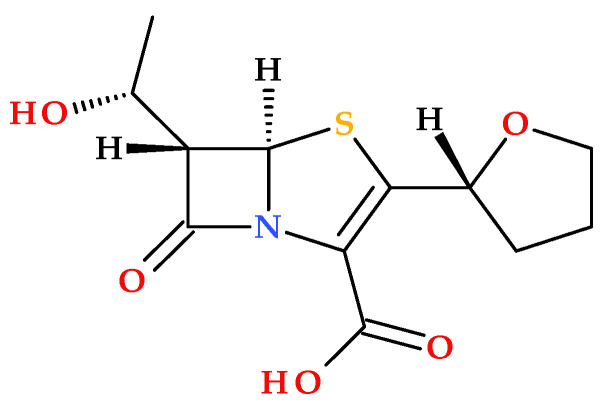
Molecular structure of faropenem (IUPAC name: (5*R*,6*S*)-6-[(1*R*)-1-hydroxyethyl]-7-oxo-3-[(2*R*)-oxolan-2-yl]-4-thia-1-azabicyclo [3.2.0]hept-2-ene-2-carboxylic acid).

Faropenem is the first penem antibiotic that was approved in Japan in 1997. It is a synthetic β-lactam antibiotic (Figure 5) [23,24,25,26].

In Japan and India, faropenem is used to treat urinary tract infections (UTIs), respiratory tract infections, skin and soft structure infections, and gynecological infections [27,28]. Faropenem sodium is also used in China, although its oral bioavailability is low [29]. The need to stop the increase in the unjustified use of faropenem sodium (especially in India and China) has been reported, given its low oral bioavailability [27,29].

**Figure 6:**
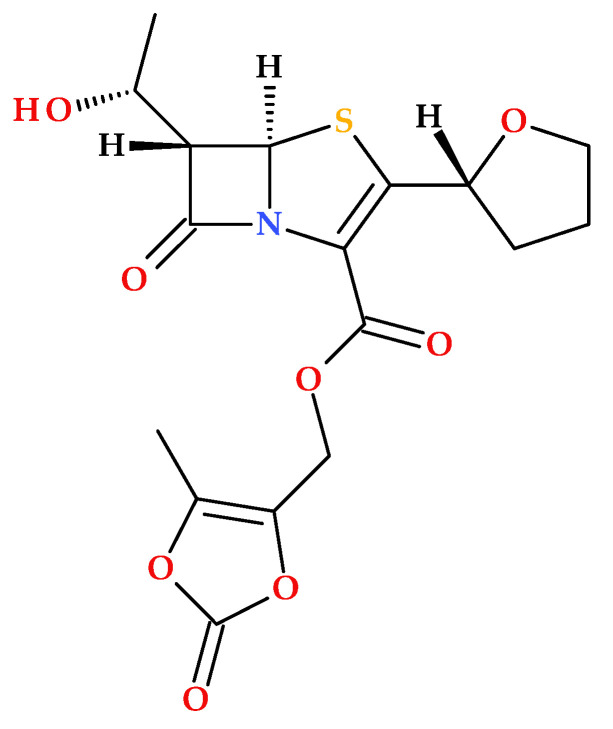
Molecular structure of faropenem medoxomil (IUPAC name: (5-methyl-2-oxo-1,3-dioxol-4-yl)methyl (5*R*,6*S*)-6-[(1*R*)-1-hydroxyethyl]-7-oxo-3-[(2*R*)-oxolan-2-yl]-4-thia-1-azabicyclo [3.2.0] hept-2-ene-2-carboxylate) [5,31].

In 2006, faropenem’s prodrug—known as faropenem medoxomil or faropenem daloxate (Figure 6)—was rejected by the FDA during phase 3 clinical trials for indications such as acute bacterial sinusitis, community-acquired pneumonia, acute exacerbations of chronic bronchitis, uncomplicated skin and soft tissue infections, and UTIs [25,30].

The Central Drugs Standard Control Organization (CDSCO) authorized the use of faropenem in India as faropenem sodium tablets in 2005, followed by an oral suspension formulation in 2021 [32].

Faropenem is missing a protonatable C3 side chain, similar to some cephalosporins or imipenem (a carbapenem antibiotic); conformational constraints on the C3 tetrahydrofuran heterocycle contribute to exceptional chemical stability and fewer associated Central Nervous System (CNS) effects, compared to imipenem [13,18].

In addition to the structural characteristics imprinted by the penem nucleus (described in the previous subchapter), it is notable that faropenem proved to be more stable than carbapenems. In addition to improving faropenem’s chemical stability, the neutral substituent at the C3 position (a chiral tetrahydrofuran ring) helps prevent hydrolysis by the kidney dehydropeptidase-I (DHP-1). One essential characteristic of penem antibiotics is their resistance to the action of bacterial or mammalian β-lactamases (as dehydropeptidases) [1,18,33]. The active metabolite of the oral prodrug faropenem medoxomil is faropenem [18]. When the carboxylic acid group of faropenem is derivatized into an ester function, faropenem’s oral bioavailability (20–30%) can be maximized. As a prodrug, faropenem exhibits a high oral bioavailability (70% to 80%) [1,13,31]. Faropenem is also stable in aqueous acid media [1,31].

Faropenem’s antimicrobial spectrum includes Gram-positive cocci, with notable efficacy against *Streptococcus pneumoniae* isolates exhibiting varying penicillin resistance. It is also active against Gram-negative bacteria, including β-lactamase-producing *Haemophilus influenzae*, *Moraxella catarrhalis*, extended-spectrum β-lactamase (ESBL)-producing *Enterobacterales*, and anaerobes [1,34,35,36,37]. However, several bacteria are not susceptible to faropenem, such as MRSA (Gram-positive bacteria), *Pseudomonas aeruginosa*, *Stenotrophomonas maltophilia*, or vancomycin-resistant *Enterococcus faecium* (Gram-negative bacteria) [18].

The safety profile of faropenem is similar to that of β-lactam antibiotics. Thereby, nausea, vomiting, and diarrhea are among the relatively rare adverse effects associated with faropenem administration [36].

### 2.3. Sulopenem

#### 2.3.1. Development and Approval

Pfizer Japan developed sulopenem (former CP-70,429) to treat uncomplicated and complicated UTIs and intra-abdominal infections [19]. After completing a comprehensive preclinical study, Pfizer Japan conducted a phase 2 trial of its intravenous formulation of sulopenem in Japan and a phase 2 trial of its oral prodrug in community-acquired pneumonia in the USA [38]. Despite the promising results, Pfizer decided to stop developing sulopenem. In 2015, Iterum Therapeutics acquired the license for sulopenem and its prodrugs and resumed the development process. Currently, three phase 3 studies are being initiated by Iterum Therapeutics to treat simple UTIs [39].

In October 2024, the FDA authorized Orlynvah (sulopenem etzadroxil and probenecid) for oral use (as tablets) to treat uncomplicated UTIs. For uncomplicated UTIs, Orlynvah is the first oral penem authorized in the USA [16,40].

#### 2.3.2. Structural Considerations

**Figure 7:**
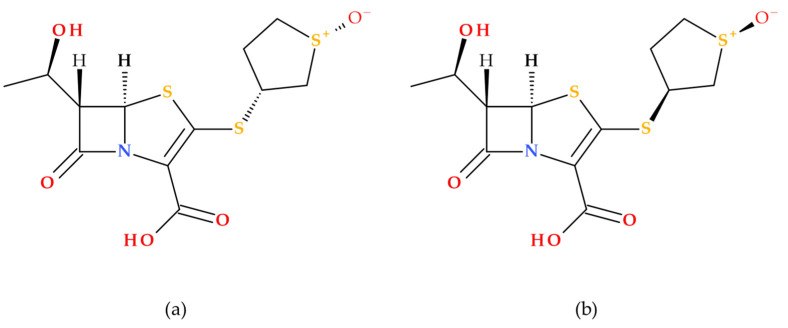
Stereochemistry of sulopenem (former CP-65,207): (**a**) CP65,207-*R* and (**b**) CP65,207-*S* [1,41].

Sulopenem’s chemical structure is similar to those of carbapenems, cephalosporins, and penicillins [19]. Sulopenem is a synthetic antibiotic produced using complete (asymmetric) synthesis; its synthesis utilizes *L*-aspartic acid to generate the chiral precursor of the C3 side chain. Structurally, sulopenem is a thiopenem [17]. Also, sulopenem (former CP-65,207) is chemically a diastereomeric combination of CP65,207-*R* and CP-65,207-*S*, two *cis*-thiophanesulfoxides (Figure 7) [1].

Similar to penams, penems are distinguished by two chiral centers: 5*R* and, depending on the type of C6 side chain (1′*R*-hydroxyethyl or acylamino substituent), 6*S* or 6*R* [41]. Early research revealed that the *S* isomer of CP-65,207 (later known as sulopenem) was more stable against the renal enzyme DHP-1, had higher drug concentrations in the urine, and was more easily absorbed than the *R* isomer. These findings opened the way for the drug’s subsequent development as a UTI antimicrobial. The prodrug sulopenem etzadroxil is hydrolyzed by intestinal esterases before absorption in the gastrointestinal tract [19].

An essential feature of the carbapenem core structure is a *trans*-α-1-hydroxyethyl substituent, which increases the resistance against various β-lactamase enzymes, including ESBLs [19,42]. Carbapenems have a *trans*-oriented side chain, unlike other groups of β-lactam antibiotics, with a *cis*-oriented side chain at the C6 position [43]. The structural feature (6-[(1*R*)-1-hydroxyethyl]) is also found in sulopenem (Figure 7). The β-lactam ring’s nitrogen atom and the thiazolidine ring’s sulfur atom enhance the carbonyl’s reactivity, transforming it into a stronger electrophile and enhancing sulopenem potency [19].

Sulopenem’s basic structure differs from those of carbapenems because it retains a sulfur atom at position 4 of the five-membered ring; therefore, it is classified as a thiopenem rather than a carbapenem. The sulfur atom can stop the degradation under the action of DHP-1 without needing a methyl group. Sulopenem’s thioether side chain has a cyclic sulfoxide group in the (*S*) configuration, which produces resonance-based positive and negative charges. It was discovered that the (*S*) configuration had a higher concentration in urine, higher absorption, and greater stability against hydrolysis. Compared to carbapenems, the β-lactam ring of sulopenem is more strained by the double bond between C2 and C3, which makes it more reactive to nucleophiles, similar to the serine in PBPs’ active site. A carboxylic moiety is also found at the core structure’s C2 position. The carboxylic moiety is necessary to obtain the prodrug sulopenem etzadroxil and to interact with lysine in the PBP active site [5,19,40].

Table 1 shows the structural differences between penems (faropenem and sulopenem) and carbapenems (drugs approved by the FDA, EMA or in the countries of origin). The molecular structures of the important carbapenems used in therapy are presented in Figure 4 (Section 2.1) [5,18,19,20,21,22]. Biapenem is approved in Japan and China, and panipenem in Japan, China, and South Korea [19,21].

**Figure 8:**
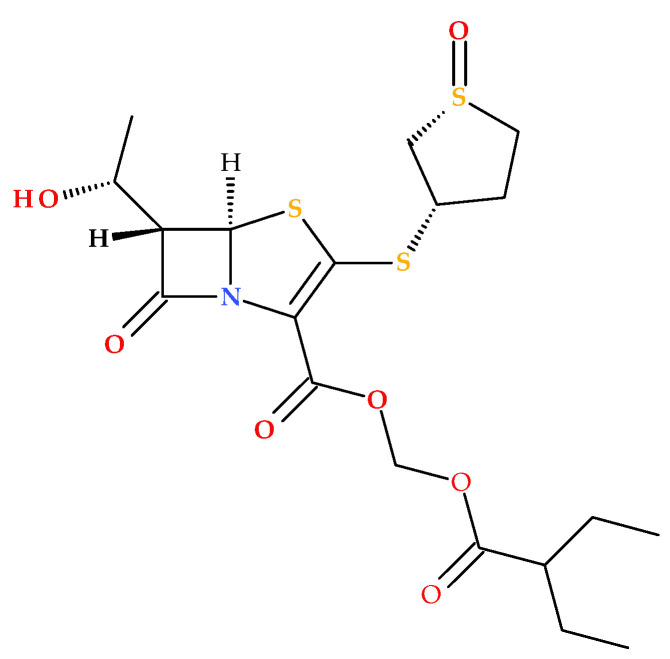
The molecular structure of sulopenem etzadroxil (IUPAC name: 2-ethylbutanoyloxymethyl (5*R*,6*S*)-6-[(1*R*)-1-hydroxyethyl]-7-oxo-3-[(1*R*,3*S*)-1-oxothiolan-3-yl]sulfanyl-4-thia-1-azabicyclo [3.2.0]hept-2-ene-2-carboxylate) [5,40,44].

The prodrug form of sulopenem is created by joining an etzadroxil group (ethyl 2-ethylbutanoate) to the carboxylic acid at the C2 position (Figure 8). The etzadroxil fragment offers stability in the oral formulation. When intestinal esterases hydrolyze it, sulopenem is released for bloodstream absorption [5,19,40].

**Figure 9:**
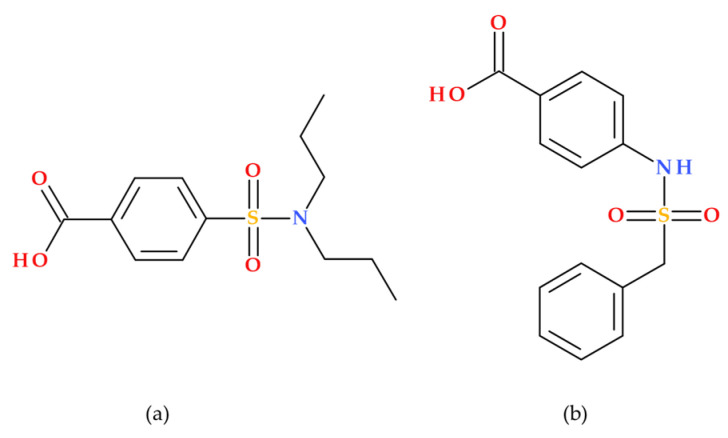
Molecular structure of (**a**) probenecid (IUPAC name: 4-(dipropylsulfamoyl)benzoic acid), and (**b**) carinamide (IUPAC name: 4-(benzylsulfonylamino)benzoic acid) [5,40].

As an inhibitor of organic anion transporters 1 and 3 (OAT1/3), probenecid (Benemid) may raise the plasma concentrations of medications whose clearance depends on OAT1/3. The chemical structure of carinamide served as the basis for developing probenecid (Figure 9), which was discovered to function similarly by competitively preventing the renal reabsorption of many organic acids. Beyer et al. (1944) reported it to lower penicillin’s renal clearance. It has been shown that probenecid raises penicillin levels in the blood. Additionally, probenecid successfully improved the retention of other antibiotics [45,46].

#### 2.3.3. Physicochemical Properties of Sulopenem

The relevant physicochemical properties of sulopenem are summarized in Table 2.

It is noteworthy that sulopenem complies with Lipinski’s rules (Ro5) [5,52]. Sulopenem does not violate any of the following criteria: a MW greater than 500 Da, a computed logP (ClogP) greater than 5, more than five hydrogen bond donors (HBD), or more than ten hydrogen bond acceptors (HBA) (atoms of nitrogen and oxygen) [53].

#### 2.3.4. Mechanism of Action

The oral prodrug formulation (sulopenem etzadroxil) is hydrolyzed by intestinal esterases to provide sulopenem’s active form [19]. Sulopenem exerts its bactericidal effect by inhibiting bacterial cell wall synthesis. It binds to PBPs in the bacterial cell membrane, preventing the cross-linking of peptidoglycan strands essential for cell wall integrity [54]. PBP serine residues are alkylated by the sulopenem’s β-lactam ring, preventing peptidoglycan cross-linking. The disruption weakens the cell wall, leading to cell lysis and death. Sulopenem can penetrate outer membrane proteins to access PBPs of Gram-negative bacteria because of its low molecular weight and ionization [19].

Sulopenem’s small molecular size and ionization enable it to pass through the outer membrane of Gram-negative bacteria, boosting its effectiveness. It is effective against multidrug-resistant (MDR) strains (see the Section 2.3.5). Despite resistance to many β-lactamases, bacterial resistance can still develop through mechanisms similar to PBP alterations, carbapenemase expression, reduced outer membrane protein expression, and efflux pumps [13,15,19].

The other component of the Orlynvah combination, probenecid, is an inhibitor of organic anion transporters 1 and 3 (OAT1/3). The role of Probenecid is to increase the plasma concentrations of sulopenem [45,46].

#### 2.3.5. Antibacterial Activity and Bacterial Resistance Mechanisms

Sulopenem exhibits an activity spectrum similar to that of ertapenem and tebipenem. Gram-negative and Gram-positive bacteria are susceptible to the effects of sulopenem [19]. *Escherichia coli*, *Klebsiella pneumoniae*, and *Proteus mirabilis* are Gram-negative bacteria against sulopenem that have been demonstrated to be effective both in vitro and in clinical infections [40]. Fluoroquinolone-resistant, ESBL-producing, MDR *Enterobacterales* are susceptible to sulopenem’s action in vitro. Early research revealed that sulopenem had in vitro action against *Streptococcus pneumoniae*, *Escherichia faecalis*, *Listeria monocytogenes*, methicillin-resistant *Staphylococcus aureus* (MSSA), and *Staphylococcus epidermidis*. It binds to PBP2, PBP1a, PBP1b, PBP4, and PBP5 in the order of highest to lowest binding affinity [19,40].

Sulopenem is ineffective against *Pseudomonas*, *Enterococcus*, MRSA, and carbapenem-resistant *Enterobacteriaceae*, similar to carbapenems. Also, some action of sulopenem against *Acinetobacter* species was demonstrated. However, sulopenem is not a first-line treatment for such infections because other carbapenems are more effective [43].

Maher J.M. et al. (2025) assessed the in vitro antimicrobial activity of sulopenem through various methods, including the post-antibiotic effect, the sub-inhibitory minimal inhibitory concentration post-antibiotic effect, checkerboard assays, and time-kill experiments. The current antimicrobial characteristics observed at concentrations near the minimum inhibitory concentration (MIC) endorse the refinement of sulopenem dosing and its continued development [14].

Sulopenem has demonstrated efficacy against several MDR bacterial strains, making it a promising option for treating resistant infections. It is effective against *Escherichia coli*, including strains that produce ESBLs and are resistant to fluoroquinolones. *Klebsiella pneumoniae*, another ESBL-producing bacterium, is also susceptible to sulopenem. *Proteus mirabilis*, a common pathogen in urinary tract infections (UTIs), also shows susceptibility to sulopenem. *Streptococcus pneumoniae*, including penicillin-resistant strains, and *Haemophilus influenzae*, including β-lactamase-producing strains, are also effectively targeted by sulopenem. These findings highlight sulopenem’s potential as a valuable treatment option for infections caused by MDR pathogens, particularly in urinary and intra-abdominal infections [19,40,55,56].

Sulopenem is resistant to many β-lactamases. However, bacterial resistance to sulopenem can be developed through several mechanisms: (a) alterations in PBPs (e.g., MRSA), (b) expression of carbapenemases (e.g., carbapenemase-producing *Enterobacterales*, *Stenotrophomonas maltophilia*), (c) a decrease in outer membrane protein expression (e.g., some *Klebsiella* spp.), (d) the existence of efflux pumps (e.g., MexAB-OprM in *Pseudomonas aeruginosa*), and (e) combined mechanisms from points (a) to (d) [19]. Thus, changes in PBPs can reduce the binding affinity of sulopenem, making it less effective. Carbapenemases, similar to metallo-β-lactamases and class A carbapenemases, can hydrolyze sulopenem, rendering it inactive. Bacteria such as *Pseudomonas aeruginosa* use efflux pumps (e.g., MexAB-OprM) to expel sulopenem from the cell, decreasing its intracellular concentration. Some bacteria reduce the expression of outer membrane proteins, limiting sulopenem’s ability to penetrate the cell. Often, resistance is due to a combination of the factors listed above, making it more challenging to overcome [14,15,19].

#### 2.3.6. Indications

A combination of sulopenem etzadroxil and probenecid is recommended for the treatment of uncomplicated UTIs caused by specific bacteria (e.g., *Escherichia coli*, *Klebsiella pneumoniae*, *Proteus mirabilis*) for adult women with minimal or no other options for oral antibiotic treatment. However, the combination is not recommended for the primary or step-down therapy for complicated UTIs or intra-abdominal infections. It is unlikely to help the patient and raises the possibility of drug-resistant bacteria developing if the drug is prescribed without a confirmed or highly suspected susceptible uncomplicated UTI. The sulopenem etzadroxil and probenecid combination is administered orally for five days, with two tablets per day; it is recommended that the medication be taken with food [16].

#### 2.3.7. Pharmacokinetics

Following oral administration, esterases hydrolyze sulopenem etzadroxil to the active ingredient, which is then hydrolyzed and dehydrogenated (primary metabolic pathway). Two inactive metabolites of sulopenem, M1a and M1b, were shown to contribute 21.8% and 43.6% of the total. Specific chemical structures for M1a and M1b metabolites are not readily available [49]. The pharmacokinetics of intravenous sulopenem seem comparable to meropenem and imipenem [21]. The most critical parameters of sulopenem are found in Table 3.

Sulopenem is excreted in feces and urine, respectively, 44.3% (26.9% unchanged) and 40.8% (3.1% unchanged) [40].

Food and probenecid enhance sulopenem etzadroxil absorption, leading to higher serum levels of sulopenem. When combined, food and probenecid further elevate the serum exposure of sulopenem [57].

#### 2.3.8. Adverse Reactions

Several clinical studies have shown that sulopenem is well tolerated [58,59,60]. The adverse effects of sulopenem in comparative phase 3 clinical trials (SURE-1, SURE-2, and SURE-3) were summarized by Zhanel et al. (2022) [19].

Regarding the combination of sulopenem–etzadroxil with probenecid (Orlynvah), the adverse reactions occurring in ≥1% of patients in the uncomplicated UTI clinical trials (1932 patients) were diarrhea (194 patients, 10%), nausea (80 patients, 4%), vulvovaginal mycotic infection (46 patients, 2%), headache (42 patients, 2%), vomiting (29 patients, 2%), and abdominal pain (22 patients, 1%) [40].

Sulopenem patients experienced more treatment-emergent adverse events (total, 24.8% versus 13.9%; associated, 17.0% versus 6.2%), which could be explained by a higher incidence of self-limited diarrhea (12.4% versus 2.5%), according to the SURE-1 trial (NCT03354598) results about the efficacy and safety of oral sulopenem etzadroxil and probenecid versus oral ciprofloxacin in the treatment of uncomplicated UTI in adult women. On both regimens, serious adverse events were comparable [61].

In the SURE-2 trial (NCT03357614), depending on uropathogen susceptibility, hospitalized adults with pyuria, bacteriuria, and signs and symptoms of a complicated UTI were randomly assigned to 5-day intravenous sulopenem followed by oral sulopenem etzadroxil with probenecid, or 5-day intravenous ertapenem followed by oral ciprofloxacin or amoxicillin–clavulanate. Sulopenem’s oral and intravenous forms were well-tolerated and exceeded the comparator favorably [59,62].

Patients with uncomplicated UTIs were randomly assigned to receive either 3 days of ciprofloxacin or 5 days of sulopenem in a phase 3 randomized trial. Moderate, self-limiting diarrhea was the only side effect more common with sulopenem. Sulopenem, when used only when necessary, might be a valuable therapy choice for patients with uncomplicated UTIs that are known or suspected to be caused by drug-resistant bacteria [58].

In the SURE-3 trial, the treatment-emergent adverse effects of sulopenem were comparable to those of ertapenem in the recent phase 3 randomized study of the two antibiotics in patients with complex intra-abdominal infections (NCT03358576). Most occurrences were mild to moderate [19,60].

#### 2.3.9. Pharmaceutical Form

Orlynvah was launched on the market by Inotherum Therapeutics [63]. The pharmaceutical product is a bilayer tablet containing 500 mg of sulopenem etzadroxil and 500 mg of probenecid (an organic anion transport inhibitor that delays renal excretion) [40,64]. When taken with probenecid, the ester prodrug sulopenem etzadroxil, created for oral administration, enhances sulopenem absorption by 62% [19,63]. Intestinal esterases hydrolyze the oral prodrug formulation to the active form of sulopenem [19].

#### 2.3.10. Other Formulations of Sulopenem

After completing preclinical studies, Pfizer Japan conducted two phase 2 clinical trials. The first, conducted in the USA (NCT00797108), evaluated the oral prodrug form of sulopenem for treating community-acquired pneumonia. The second trial, conducted in Japan, focused on the intravenous formulation of sulopenem [38,39,65].

### 2.4. Comparison Between Penems and Carbapenems

Among β-lactam antibiotics, penems and carbapenems represent structurally related but pharmacologically distinct subclasses. While both share a β-lactam core and broad-spectrum antibacterial activity, their differences in ring structure, stability to enzymatic degradation, and clinical applications underscore the need for a comparative evaluation. Table 4 compares penems and carbapenems, highlighting stability to DHP-1, oral bioavailability, resistance profiles, and therapeutic roles in emerging multidrug-resistant pathogens.

## 3. Penems Under Development

Numerous derivatives of the penems have been synthesized and characterized. In our paper, most studies published after 2000 were identified; the reported penem analogues are summarized and briefly presented below in approximate chronological order of publication.

### 3.1. MEN 10700 and MEN 11505

**Figure 10:**
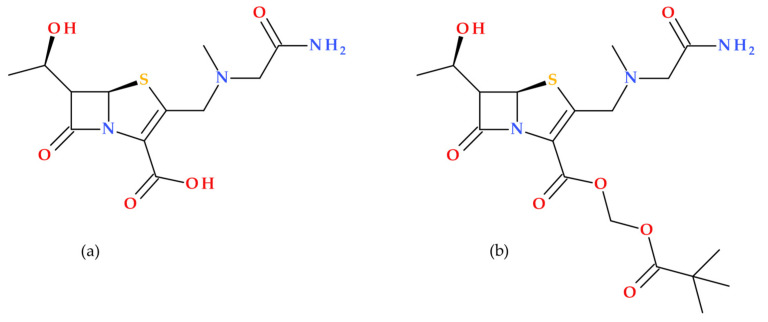
Molecular structure of (**a**) MEN 10700 (IUPAC name: (6*S*)-3-[[(2-amino-2-oxoethyl)-methylamino]methyl]-6-[(1*R*)-1-hydroxyethyl]-7-oxo-4-thia-1-azabicyclo [3.2.0]hept-2-ene-2-carboxylic acid), and (**b**) MEN 11505 (IUPAC name: 2,2-dimethylpropanoyloxymethyl (5*R*)-3-[[(2-amino-2-oxo-ethyl)-methyl-amino]methyl]-6-[(1*R*)-1-hydroxyethyl]-7-oxo-4-thia-1-azabicyclo [3.2.0]hept-2-ene-2-carboxylate) [5,67,69].

MEN 10700 is an innovative penem antibiotic developed at Menarini Spa in Florence, Italy. It features a unique alkylpenem structure with a sarcosinamido group at the C3 position (Figure 10) [66]. Arcamone F.M. et al. (2002) presented in their study the synthesis and antibacterial activity of the penem compound MEN 10700. The new antibiotic exhibited broad-spectrum antibacterial activity and was effective against Gram-positive and Gram-negative, anaerobic, and resistant bacteria. MEN 10700 was resistant to degradation by renal DHP-1 enzymes [67]. Previously, it was demonstrated that MEN 10700 outperforms ritipenem and faropenem in various aspects, making it a compelling alternative to carbapenems, especially when antipseudomonal activity is not crucial, and the oral treatment is preferred [66].

In addition, in vitro antibacterial activity on clinical isolates was determined by Ferrari L. et al. (2002). Generally, MEN 10700 exhibited activity comparable to that of imipenem and meropenem. MEN 10700 showed high efficacy against MRSA and *Staphylococcus epidermidis*, making it the most potent among tested antibiotics. However, it was less effective than carbapenem antibiotics against *Morganella morganii*, *Serratia marcescens*, and *Acinetobacter* species. Ciprofloxacin-resistant *Escherichia coli* were highly susceptible to MEN 10700, imipenem, and meropenem, with much lower susceptibility to other antibiotics such as cefepime. This trend was more pronounced in ESBL-producing strains of the KES group (*Klebsiella*, *Enterobacter*, and *Serratia*), where MEN 10700, imipenem, and meropenem showed significantly lower MIC90 values compared to other antibiotics [68].

MEN 10700 was optimized as an oral prodrug, pivaloyloxymethyl ester (MEN 11505). The molecular structures of MEN 10700 and MEN 11505 are presented in Figure 10 [67,68].

#### 3.1.1. Stability, Metabolism and Side Effects

MEN 10700 is known for its high stability against enzymatic degradation, particularly by renal dehydropeptidase DHP-1. The stability against enzymatic degradation is essential for maintaining its antibacterial efficacy. MEN 11505 is the orally absorbed ester prodrug of MEN 10700; it also demonstrates high stability against enzymatic degradation, ensuring that it can effectively release MEN 10700 for absorption [67].

MEN 10700 exhibits a broad antibacterial spectrum against Gram-positive and Gram-negative bacteria [66,70]. It is effective against anaerobes and strains resistant to other antibiotics [67]. The conversion of MEN 11505 into MEN 10700 is efficient, with a relative bioavailability of 43% in rats. A significant amount of the MEN 11505 administered dose is converted into the active form, making it adequate for oral use [67].

The adverse effects of MEN 10700 are generally consistent with those of other β-lactam antibiotics. Common side effects may comprise gastrointestinal disturbances, allergic reactions, and potential nephrotoxicity [66,70]. However, the literature does not extensively document detailed adverse effect profiles for MEN 10700. Adverse effects of MEN 11505 are likely similar to those of MEN 10700 once it is metabolized. The literature does not provide extensive specific adverse effect data for MEN 11505, but it can be inferred that it shares the same risk profile as MEN 10700 [67]. No specific clinical studies directly focus on MEN 10700 or MEN 11505.

#### 3.1.2. Limitations

MEN 10700, despite its broad antibacterial spectrum, is less effective against certain strains such as *Pseudomonas aeruginosa*, *Stenotrophomonas maltophilia*, and *Enterococcus faecium*, and faces the risk of bacterial resistance over time, which could limit its long-term efficacy [66,67]. While it shows resistance to degradation by renal DHP-1 enzymes and outperforms ritipenem and faropenem in various aspects, it is less effective than carbapenem antibiotics against *Morganella morganii*, *Serratia marcescens*, and *Acinetobacter species* [68]. Additionally, its antibacterial spectrum is slightly narrower than other antibiotics (e.g., imipenem). On the other hand, MEN 11505, the orally absorbed ester of MEN 10700, has a relative bioavailability of 43% after oral administration in rats, meaning less than half of the administered dose is effectively absorbed. Although MEN 11505 shows high stability against enzymatic degradation, this stability might not be sufficient in all clinical scenarios, and it also faces challenges related to the development of bacterial resistance [66,67]. The existing limitations underscore the need for ongoing research and development to enhance the efficacy and safety of MEN 10700 and MEN 11505.

### 3.2. Imidazole Substituted 6-Methylidene-Penems

**Figure 11:**
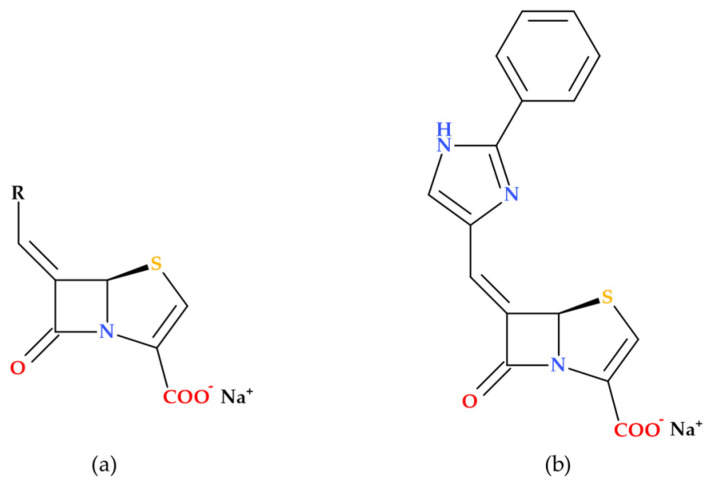
(**a**) General molecular structure of imidazole substituted 6-methylidene-penem derivatives as potent β-lactamase inhibitors, and (**b**) molecular structure of (5*R*),(6*Z*)-6-(2-benzyl-1*H*-imidazol-4-yl-methylene)-7-oxo-4-thia-1-aza-bicyclo [3.2.0]hept-2-ene-2-carboxylic acid sodium salt) [71].

Based on modeling studies, Venkatesan A. et al. (2004) designed and synthesized several novel imidazole-substituted 6-methylidene-penem derivatives, which were tested as potent inhibitors against various β-lactamase-producing bacterial isolates (Figure 11). The new compounds demonstrated outstanding in vitro activity against both class A and class C enzymes, and their spectrum of activity is broader than any inhibitors currently on the market. Five obtained compounds were tested in combination with piperacillin in antimicrobial susceptibility assays. Isolates of *Escherichia coli* (11), *Escherichia cloacae* (3), *Klebsiella pneumoniae* (1), *Serratia marcescens* (2), *Pseudomonas aeruginosa* (1), *Stenotrophomonas maltophilia* (1), and *Staphylococcus aureus* (1) bacterial strains were used. A percentage of 90% of the bacterial strains in the study were sensitive to piperacillin when associated with (5*R*),(6*Z*)-6-(2-benzyl-1*H*-imidazol-4-yl-methylene)-7-oxo-4-thia-1-aza-bicyclo [3.2.0]hept-2-ene-2-carboxylic acid sodium salt, a 2-benzyl imidazole derivative (Figure 11). In vivo, the same compound increased piperacillin’s activity against *Escherichia coli* LSU 80-8, specifically targeting the TEM-1 enzyme [71].

The research revealed that when used independently, the new imidazole-substituted 6-methylidene-penem derivatives lack inherent antibacterial activity. However, when combined with piperacillin, the new compounds substantially lower the MIC values for various piperacillin-resistant bacteria, demonstrating improved antibacterial effectiveness in combination with the antibiotic [71].

### 3.3. 5,5,6-Fused Tricyclic-6-Methylidene Penems

**Figure 12:**
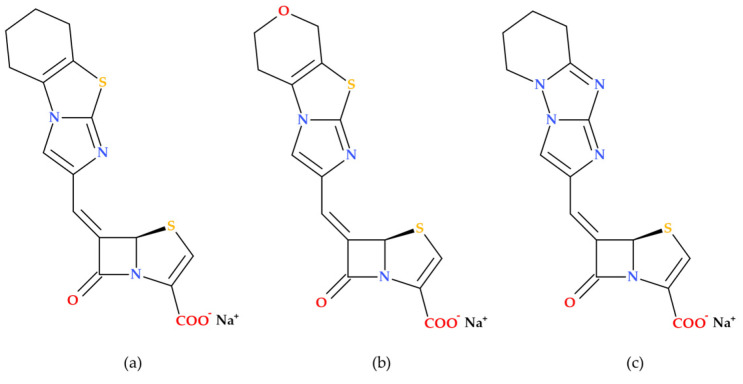
Molecular structure of some 5,5,6-fused tricyclic-6-methylidene penems: (**a**) (5*R*),(6*Z*)-7-oxo-6-(5,6,7,8-tetrahydroimidazo [2,1-*b*][1,3]benzothiazol-2-ylmethylene)-4-thia1-azabicyclo [3.2.0]hept-2-ene-2-carboxylic acid sodium salt, (**b**) (5*R*),(6*Z*)-6-(5,8-dihydro-6*H*-imidazo [2,1-*b*]pyrano [4,3-*d*][1,3]thiazol-2-ylmethylene)-7-oxo-4-thia-1-azabicyclo [3.2.0]hept-2-ene-2-carboxylic acid sodium salt, and (**c**) (5*R*),(6*Z*)-6-(4,5,6,7-tetrahydro-1,3*a*,3*b*,8-tetraaza-cyclopenta[a]indene-2-ylmethylene)-7-oxo-4-thia-1-aza-bicyclo [3.2.0]hept-2-ene-2-carboxylic acid sodium salt [72].

Venkatesan A.M. et al. (2008) report on the synthesis and evaluation—both in vitro and in vivo—of novel 5,5,6-fused tricyclic heterocycles linked to the 6-methylidene penem core as a continuation of their 2004 study aimed at further exploring structure–activity relationships (SAR). Overall, the study on novel imidazole-substituted 6-methylidene-penem derivatives offers promising directions for future drug development, particularly in the design of potent and broad-spectrum β-lactamase inhibitors. The penem derivatives discussed in this paper are potent broad-spectrum TEM-1 and AmpC β-lactamase inhibitors. In combination with piperacillin, their in vitro activities demonstrated higher susceptibility to the class A and C-resistant strains of bacteria that were investigated. In the acute fatal infection model, three synthesized compounds (Figure 12) showed in vivo action against TEM-1-producing pathogens [72].

#### Essential Elements of Structure-Activity Relationship (SAR) Study

Several aspects of SAR are highlighted by Venkatesan A.M. et al. (2008), including substituent effects, inhibitory activity, and antibacterial activity [72]. The primary observed effects were associated with variations at the 2-position, specifically in comparing 2-phenyl to 2-benzyl substitution, as well as 2-thiazole and N-methyl imidazole substituents.

- The 2-benzyl-substituted imidazole derivative ((5*R*),(6*Z*)-6-(2-benzyl-1*H*-imidazol-4-yl-methylene)-7-oxo-4-thia-1-aza-bicyclo [3.2.0]hept-2-ene-2-carboxylic acid sodium salt) exhibited significantly higher potency compared to the 2-phenyl-substituted derivative ((5*R*),(6*Z*)-6-(2-phenyl 1*H*-imidazol-4-yl-methylene)-7-oxo-4-thia-1-aza-bicyclo [3.2.0]hept-2-ene-2-carboxylic acid sodium salt). The aspect is attributed to the enhanced π-π stacking interaction with Tyr 105 in the TEM-1 active site;
- The 2-thiazole-substituted derivative ((5*R*),(6*Z*)-6-(2-thiazol-2-yl-1*H*-imidazol-4-yl-methylene)-7-oxo-4-thia-1-aza-bicyclo [3.2.0]hept-2-ene-2-carboxylic acid sodium salt) showed moderate potency. Methylation of the imidazole nitrogen ((5*R*),(6*Z*)-6-(1-methyl-2-thiazol-2-yl-1*H*-imidazol-4-yl-methylene)-7-oxo-4-thia-1-aza-bicyclo [3.2.0]hept-2-ene-2-carboxylic acid sodium salt) did not significantly alter the potency against TEM-1, but improved activity against AmpC;
- The N-methyl imidazole derivative ((5*R*),(6*Z*)-6-(3,10-dimethyl-3*H*,10*H* [2,40]bi-imidazolyl-4-yl-methylene)-7-oxo-4-thia-1-azabicyclo [3.2.0]hept-2-ene-2-carboxylic acid sodium salt) demonstrated increased potency against TEM-1 and AmpC enzymes but was less effective against Imi-1.

All newly synthesized compounds were found to be potent inhibitors of both class-A and class-C enzymes. Also, the study found that these penem derivatives do not exhibit inherent antibacterial activity when tested alone. Thus, when combined with piperacillin, the penem derivatives significantly reduce the MIC values (compared to piperacillin alone) against various piperacillin-resistant bacteria, indicating enhanced antibacterial activity in combination with the antibiotic [72], similar to compounds from the previous study [71]. The combination of (5*R*),(6Z)-6-(2-benzyl-1*H*-imidazol-4-yl-methylene)-7-oxo-4-thia-1-aza-bicyclo [3.2.0]hept-2-ene2-carboxylic acid sodium salt and piperacillin was particularly effective, rendering 90% of the tested organisms susceptible [72].

### 3.4. Penem Inhibitors of Bacterial Signal Peptidase

**Figure 13:**
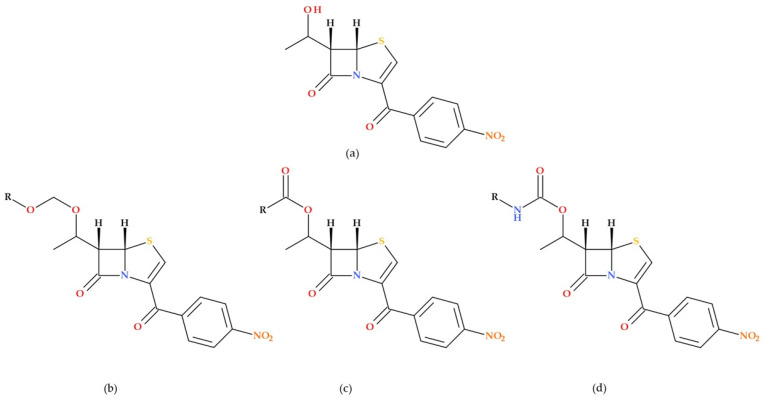
General molecular structure of penem derivatives: (**a**) the parent compound (5*S*,6*S*)-6-(1-hydroxyethyl)-2-(4-nitrobenzoyl)-4-thia-1-azabicyclo [3.2.0]hept-2-en-7-one, (**b**) methoxy/ethoxy methyl ether derivatives (R = methyl or ethyl), (**c**) isopropyl derivative (R = isopropyl), and (**d**) carbamate derivatives (R = ethyl or isopropyl) [73].

Signal peptidase is a crucial enzyme in Gram-positive and Gram-negative bacteria, which is required to process cell surface-bound preproteins. Harris D.A. et al. (2009) discuss the synthesis of 5*S* penems from 6-aminopenicillanic acid and their biological evaluation as inhibitors of bacterial type I signal peptidase. Their unique catalytic mechanism makes them an attractive target for novel antibiotics. The synthesized 5*S* penems showed in vivo activity and were evaluated against several bacteria, including *Escherichia coli*, *Staphylococcus aureus*, MRSA, and *Staphylococcus epidermidis*. SAR studies were conducted to optimize the penem for better signal peptidase binding. Modifications at the C6 position of the penem core were explored to enhance its binding affinity to signal peptidase. The parent compound had a 5*S*,6*S*-penem core with a C3 *p*-nitrobenzyl-protected carboxylic acid and a C6 hydroxyethyl moiety (Figure 13). Four synthesized penems showed activity against *Staphylococcus epidermidis* with MIC values of 50 µg/mL (parent compound, methoxy methyl ether derivative, ethoxy methyl ether derivative, and isopropyl derivative). Two synthesized penems (carbamate derivatives) showed modest activity against *Staphylococcus epidermidis* and MRSA with a 100 µg/mL MIC. The study highlights the potential of penem inhibitors as antibiotics with novel mechanisms of action [73].

### 3.5. BLI-489

**Figure 14:**
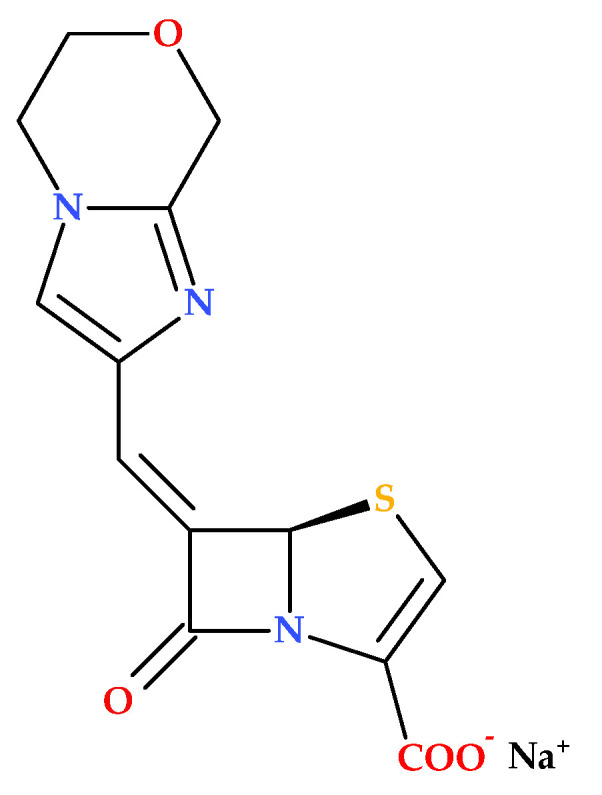
The molecular structure of BLI-489 (IUPAC name: (5*R*,6*Z*)-6-(6,8-dihydro-5*H*-imidazo [2,1-c][1,4]oxazin-2-ylmethylidene)-7-oxo-4-thia-1-azabicyclo [3.2.0]hept-2-ene-2-carboxylic acid sodium salt) [5,74].

BLI-489 emerged as part of continuing studies to fight antibiotic resistance, especially against bacteria that produce β-lactamases. Structurally, BLI-489 is a bicyclic-6-methylidene penem molecule (Figure 14). It was previously reported that attaching bicyclic and tricyclic groups to the C6 position of the penem molecule via a methylidene bond results in inhibitory activity against class A, C, and D β-lactamase enzymes. Therefore, it was shown that BLI-489 can inhibit class A, C, and D β-lactamase enzymes and other β-lactamases [74].

Petersen P.J. et al. (2009) investigated the effectiveness of BLI-489 in combination with piperacillin against various bacterial strains. The study focuses on developing reliable in vitro testing methods to evaluate the antimicrobial activity of BLI-489 in combination with piperacillin. Key findings include the identification of optimal ratios and concentrations for accurate susceptibility testing and the comparative efficacy of the piperacillin-BLI-489 combination against different classes of β-lactamase-producing bacteria. The research highlights the potential and limitations of BLI-489, emphasizing the need for further optimization and validation in clinical settings. Thus, BLI-489 faces several limitations. In vitro, susceptibility testing revealed inconsistencies, with specific ratios of piperacillin-BLI-489 leading to false reports of susceptibility or intermediate resistance. At a constant concentration, BLI-489 tended to overpredict resistance, potentially excluding effective treatments. The compound also did not significantly enhance the activity of piperacillin against enterococci, indicating limited efficacy across all bacterial strains [74].

Studies have highlighted BLI-489 as a promising β-lactamase inhibitor with potential effectiveness in combination therapies to combat antibiotic-resistant bacteria [75,76].

The synergistic effects of BLI-489 and imipenem against carbapenem-resistant *Acinetobacter baumannii* were assessed by Wang Y.-C. et al. (2021). The study demonstrated that the combination significantly inhibited OXA-23, OXA-24, OXA-51, and OXA-58 β-lactamases produced by carbapenem-resistant *Acinetobacter baumannii*. Despite showing promising synergistic effects with imipenem against certain β-lactamase-producing strains, its effectiveness varied significantly, particularly against OXA-23-producing isolates. In vivo studies demonstrated inconsistent results, suggesting that the compound’s efficacy might be influenced by factors not fully understood or controlled in preclinical models. Additionally, there is a risk of resistance development over time, and the specificity and binding affinity of BLI-489 to different β-lactamases could limit its broad-spectrum applicability. As a preclinical compound, its safety, efficacy, and pharmacokinetics in humans remain unverified, necessitating extensive clinical trials to confirm its potential as a viable treatment option [75].

In another study, Shi S. et al. (2022) examined the antibacterial efficacy of BLI-489 combined with imipenem or meropenem against carbapenem-resistant *Enterobacterales* pathogens. As a novel β-lactamase inhibitor, BLI-489 may offer an alternative to treating clinical infections caused by strains of bacteria resistant to carbapenem [76]. However, the study by Shi S. et al. (2022) includes the limited scope of strains tested, which may not fully represent the diversity of carbapenemase-producing carbapenem-resistant *Enterobacterales* in clinical settings, and the use of in vitro and in vivo models that do not perfectly mimic human infections. Additionally, the long-term safety and potential side effects of BLI-489 in humans remain unknown, and the study does not address the potential for resistance development against BLI-489. The effectiveness of BLI-489 relies on its combination with imipenem or meropenem, and its efficacy with other antibiotics was not explored [76].

BLI-489 has completed four phase 1 clinical trials, registered on ClinicalTrials.gov—NCT00820404 [77], NCT00894439 [78], NCT00854009 [79], and NCT00909688 [80].

However, there are several question marks regarding the development of BLI-489 as a drug. In susceptibility testing, specific methodologies using BLI-489 can lead to the overprediction of resistance in bacterial strains. The in vitro activities of the antibiotics were assessed against aerobic and anaerobic bacteria using the broth microdilution and agar dilution methods, respectively, as recommended by the Clinical and Laboratory Standards Institute (CLSI) [74]. While BLI-489 synergizes with other antibiotics, its effectiveness may differ based on the bacterial strain and the type of carbapenemase produced [76]. Moreover, BLI-489 encounters typical preclinical challenges, such as translating positive in vitro results to in vivo efficacy. Animal models may not always accurately predict human responses, and the high costs and long duration of preclinical studies add to the complexity. The limitations underscore the need for further optimization and testing before BLI-489 can be considered a reliable therapeutic option. Being a synthetic compound, BLI-489 carries inherent risks in research and development, including potential unforeseen side effects or limitations in practical applications. In this regard, further clinical studies are necessary.

### 3.6. Ferrocene-Containing Penems

The ferrocenyl group’s stability, non-toxicity, ease of membrane permeation, and favorable electrochemical properties, along with the wide variety of accessible derivatives, have made ferrocene and its derivatives highly suitable for biological applications and for conjugation with biomolecules (e.g., antibacterial, antimalarial, anti-coronavirus, antitumor, and antimycotic activity) [81,82,83,84,85].

**Figure 15:**
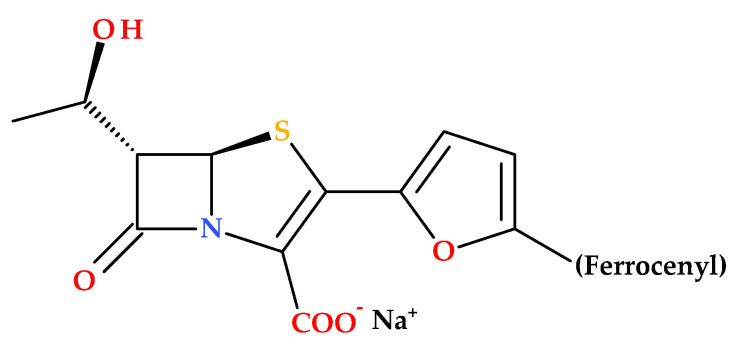
Molecular structure of (5*R*,6*S*)-6-((*R*)-l-hydroxyethyl)-2-(5-ferrocenyl-2-furyl)penem-3-carboxylic acid sodium salt [85].

Long B. et al. (2010) described the synthesis and SAR of new penem compounds with a ferrocenyl group at the C2 position. The new penem derivatives were characterized as sodium salts by spectroscopic methods (^1^H NMR, IR) and elemental analysis. The compounds were tested for antibacterial activities against Gram-positive (including MRSA) and Gram-negative bacteria. Most penems showed superior or equivalent antibacterial efficacy and high stability against DHP-1 compared to faropenem. Notably, the compound (5*R*,6*S*)-6-((*R*)-l-hydroxyethyl)-2-(5-ferrocenyl-2-furyl)penem-3-carboxylic acid sodium salt (Figure 15) was especially effective against diverse Gram-positive and Gram-negative bacteria (MRSA, *Serratia marcescens* IAM 1136, *Pseudomonas aeruginosa* PAO-1, *Bacillus subtilis* ATCC 6633, *Escherichia coli* NIHJ JC-2, *Proteus vulgaris* GN 7919, *Citrobacter freundii* GN 7391 (β-lactamase-producing organism) [85].

The redox properties of ferrocene play an essential role in enhancing the antimicrobial activity of ferrocene-containing penems. Ferrocene redox properties contribute to the generation of ROS, the disruption of cellular redox homeostasis, and increased stability against enzymatic degradation. As a result, ferrocene-containing penems exhibit potent antibacterial activity against a broad spectrum of pathogens, including resistant strains [85].

### 3.7. Bicycle Substituted 6-Methylidene-Penems

**Figure 16:**
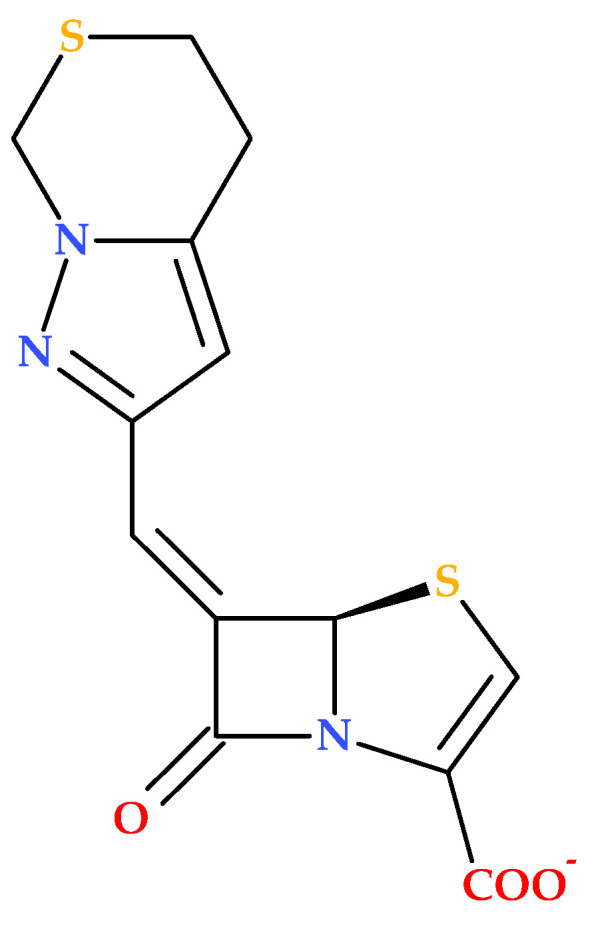
Molecular structure of (5*R*,6*Z*)-6-(5,7-dihydro-4*H*-pyrazolo [1,5-c][1,3]thiazin-2-ylmethylene)-7-oxo-4-thia-1-azabicyclo [3.2.0]hept-2-ene-2-carboxylate [86].

The study conducted by Hazra S. et al. (2015) investigates the interaction of two penem derivatives (provided by Pfizer) with the β-lactamase enzyme from *Mycobacterium tuberculosis*. The BlaC enzyme exhibits strong activity against penicillins and cephalosporins, but its activity against carbapenems is comparatively weaker. The compound (5*R*,6*Z*)-6-(5,7-dihydro-4*H*-pyrazolo [1,5-*c*][1,3]thiazin-2-ylmethylene)-7-oxo-4-thia-1-azabicyclo [3.2.0]hept-2-ene-2-carboxylate (sodium) (Figure 16) was found to inhibit BlaC more efficiently than clavulanate [86].

In addition, the discussed penem derivative forms a covalent complex with BlaC, leading to significant growth inhibition of *Mycobacterium tuberculosis*; this suggests that the bicycle-substituted 6-methylidene-penem inhibits BlaC and affects peptidoglycan cross-linking transpeptidases, enhancing its antibacterial properties [86].

### 3.8. T405 and Derivatives

**Figure 17:**
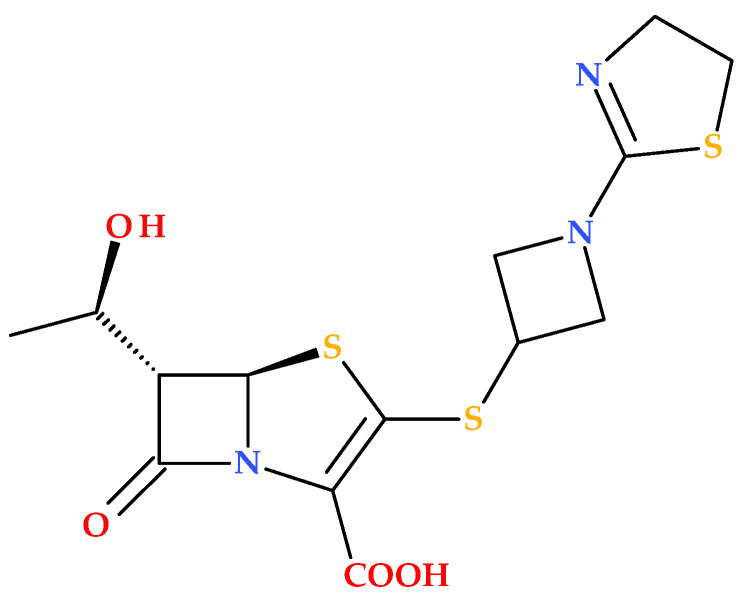
Molecular structure of T405: (5*S*,6*R*)-3-((1-(4,5-dihydrothiazol-2-yl)azetidin-3-yl)thio)-6-((*R*)-1-hydroxyethyl)-7-oxo-4-thia-1-azabicyclo [3.2.0]hept-2-ene-2-carboxylic acid [87].

Batchelder H.R. et al. (2020) conducted a study regarding the T405 compound, a new β-lactam penem synthesized in-house (Figure 17) [87].

**Figure 18:**
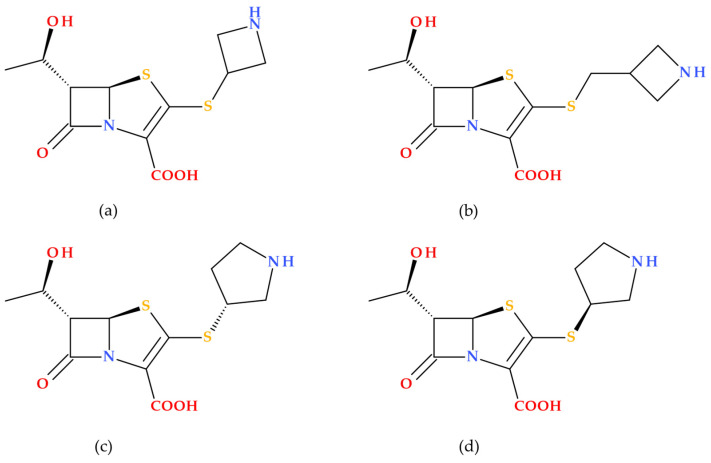
Molecular structure T405 derivatives: (**a**) T422: (5*R*,6*S*)-3-(azetidin-3-ylthio)-6-((*R*)-1-hydroxyethyl)-7-oxo-4-thia-1-azabicyclo [3.2.0]hept-2-ene-2-carboxylic acid, (**b**) T428: (5*R*,6*S*)-3-((azetidin-3-ylmethyl)thio)-6-((*R*)-1-hydroxyethyl)-7-oxo-4-thia-1-azabicyclo [3.2.0]hept-2-ene-2-carboxylic acid, (**c**) T425: (5*R*,6*S*)-6-((*R*)-1-hydroxyethyl)-7-oxo-3-(((*R*)-pyrrolidin-3-yl)thio)-4-thia-1-azabicyclo [3.2.0]hept-2-ene-2-carboxylic acid, and (**d**) T426: (5*R*,6*S*)-6-((*R*)-1-hydroxyethyl)-7-oxo-3-(((*S*)-pyrrolidin-3-yl)thio)-4-thia-1-azabicyclo [3.2.0]hept-2-ene-2-carboxylic acid [88].

T405 demonstrated potent activity against *Mycobacteroides abscessus* and drug-resistant strains from cystic fibrosis patients. The resistance to T405 was almost undetectable in combination with avibactam (a non-β-lactam β-lactamase inhibitor). Additionally, T405 exhibited a favorable pharmacokinetic profile and no toxicity at high doses in mice [87]. Subsequently, a library of penem compounds with C3 side-chain variations was synthesized, and their antimicrobial activities were tested against *Mycobacterium tuberculosis* H37Rv and *Mycobacteroides abscessus* ATCC 19977. Several new penems displayed more potent antimicrobial activity than standard carbapenem antibiotics, with some surpassing the efficacy of T405. Additionally, many of the new compounds showed minimal or no increase in the MIC in the presence of serum compared to the highly protein-bound T405. The study highlighted four derivatives (T422, T425, T426, and T428) (Figure 18) as compounds with low MICs against MtbH37Rv and low plasma protein binding (PPB). The MIC data of tested analogues indicate the azetidine ring’s critical role in the candidates’ activity. At the same time, changes to the 2-thiazoline were either detrimental or had no impact. Additionally, in vitro analysis revealed that the side chain did not affect adduct stability with *L*,*D*-transpeptidases, but it did influence the adduct stability with PBPs. These aspects suggest that the variations in side-chain activity depend more on the inhibition of PBPs [88].

The essential SARs established in the study of Batchelder H. et al. (2022) are presented further. The penem core structure was crucial for maintaining activity, with modifications to the side chains influencing the overall effectiveness. The study synthesized a library of penems with different C3 side chains, focusing on the azetidine ring and 2-thiazoline ring modifications. Increasing the size of the azetidine ring maintains or improves antimicrobial activity. For example, five-membered rings (T425 and T426) showed intense activity against *Mycobacterium tuberculosis* and *Mycobacteroides abscessus*. Aromatic rings in the 2-thiazoline-ring position generally reduced antimicrobial activity. However, non-aromatic modifications showed better results. The stability of penem adducts with Ldt_Mt2_ and DacB2 enzymes (a transpeptidase and a PBP) varied, with some penems showing long-lasting inhibition, which is desirable for treating slow-growing bacteria such as Mycobacterium tuberculosis. Penems with lower plasma protein binding showed better activity in the presence of serum, indicating their potential for in vivo efficacy [88]. These findings highlight the importance of specific structural modifications in enhancing the antimicrobial activity of penem antibiotics against mycobacteria.

Rimal B. et al. (2022) examined the efficacy of T405 for treating *Mycobacteroides abscessus* infections in mice. T405 demonstrated bactericidal properties comparable to the standard antibiotic imipenem (carbapenem antibiotic) and sustained its effectiveness over 4 weeks. Additionally, T405 displayed synergistic effects when used with other antibiotics (imipenem, cefditoren) and a β-lactamase inhibitor (avibactam). T405 is an appropriate candidate for additional preclinical research to treat *Mycobacteroides abscessus* infections due to its effectiveness when compared to imipenem and its synergism with imipenem and cefditoren. The study also outlines a method for the industrial-scale synthesis of T405, making it a promising candidate for further evaluation in treating *Mycobacteroides abscessus* infections [89].

T405 exhibits potent bactericidal activity against *Mycobacteroides abscessus* from the onset of treatment. It has a lower MIC against *Mycobacteroides abscessus* clinical isolates (1 to 8 μg/mL) compared to imipenem (32 μg/mL) and cefoxitin (64 μg/mL), indicating higher in vitro potency. T405 demonstrates stability in vivo, with its MIC remaining unaltered after 4 weeks of exposure in mouse lungs. It also shows favorable pharmacokinetics, staying in the body longer than other antibiotics, which may allow for less frequent dosing. T405 exhibits a favorable toxicity profile at elevated doses in mice, with no observable adverse effects or irritation at the injection site. Overall, T405 appears to be a promising candidate with higher efficacy and stability than some established carbapenems, and it has a favorable toxicity profile. However, further preclinical and clinical assessments are needed to demonstrate its potential fully [87,88,89].

There are several challenges in developing T405 as a drug. T405 has not undergone extensive clinical trials to confirm its safety and efficacy in humans. Most data available are from preclinical studies, primarily in mouse models. Additional extensive clinical trials are required to verify its effectiveness and safety in human patients. Although T405 has shown resistance to β-lactamase enzymes produced by *Mycobacteroides abscessus*, there is always a risk that bacteria could develop resistance over time, especially with widespread use. T405 may need to be administered with other antibiotics or β-lactamase inhibitors to enhance its efficacy; this aspect can complicate treatment regimens and increase the risk of adverse drug interactions. Similar to other treatments for *Mycobacteroides abscessus* infections, T405 may require long-term administration, which can be logistically challenging and burdensome for patients. While T405 has shown a favorable toxicity profile in mice, its long-term safety in humans remains unknown. Unforeseen side effects or toxicity issues could emerge during clinical trials.

## 4. Considerations Regarding the Future of Penems

The future of penems appears promising, given recent advancements and their critical role in combating bacterial resistance. Penem antibiotics, particularly the newly approved sulopenem (the combination of sulopenem etzadroxil with probenecid), represent significant strides in addressing MDR infections [19,43]. The particular structural characteristics of penems, including their stability against β-lactamase enzymes and effectiveness against a broad spectrum of bacteria, make them a vital asset in the antimicrobial arsenal [9,33].

Ongoing research continues to explore penem derivatives, with ongoing developments aimed at enhancing their pharmacokinetic properties and minimizing adverse effects. As new penems and their prodrug formulations (e.g., MEN 11505, faropenem medoxomil, faropenem daloxate, sulopenem etzadroxil) are synthesized and tested, the potential for more effective and accessible treatments for bacterial infections grows, offering hope in the fight against resistant pathogens [25,30,40,64,67]. The continued development of penem antibiotics holds promise for their application in a broader range of clinical settings. Efforts to optimize the structure and activity of penems will likely yield antibiotics with improved efficacy and broader antibacterial coverage (see Chapter 3).

Future studies will focus on enhancing the oral bioavailability of penems, making them more convenient and effective for outpatient treatments. For example, MEN 11505, an oral prodrug of MEN 10700, was optimized to improve bioavailability while maintaining antibacterial activity [67]. Also, a SAR study was conducted to optimize the penem for better signal peptidase binding, with modifications at the C6 position enhancing binding affinity. Thus, the study highlights the potential of penem inhibitors as antibiotics with novel mechanisms of action [73].

Some penem derivatives have shown potential as potent β-lactamase inhibitors [71,72]. SAR studies of novel 5,5,6-fused tricyclic heterocycles attached to the 6-methylidene penem core suggest that optimizing the substituents at the 2-position of the imidazole ring can significantly enhance the potency and spectrum of activity. Future research may explore additional substituents that could enhance interactions with target enzymes. The enhanced activity of the obtained derivatives in combination with piperacillin highlights the potential for developing combination therapies, and can help overcome resistance in piperacillin-resistant strains. This demonstrates the feasibility of designing broad-spectrum β-lactamase inhibitors. The insights gained can guide the development of compounds that inhibit a wide range of β-lactamase enzymes. In addition, the SAR findings provide mechanistic insights into the interactions between the inhibitors and β-lactamase enzymes. Also, the insights gained can support the rational design of novel inhibitors with enhanced potency and minimized resistance [72].

For example, BLI489 demonstrated its ability to inhibit class A, C, and D β-lactamase enzymes. The synergistic effect of BLI-489 in combination with imipenem and meropenem was also highlighted [75,76]. Additionally, the potential for combination therapies involving penems and β-lactamase inhibitors could extend their utility against resistant bacterial strains [89]. With ongoing innovation and clinical trials, penems could play a pivotal role in the future landscape of antibiotic therapy.

Penems are being explored for their efficacy against multidrug-resistant Gram-negative bacteria. Such pathogens often produce enzymes such as AmpC, ESBLs, and carbapenemases, which confer resistance to many antibiotics. Penems’ unique structural characteristics make them promising candidates for overcoming resistance mechanisms [19,55,56]. Ongoing investment in preclinical and clinical research is essential to help comprehend the treatment patterns, resistance mechanisms, and potential cross-resistance of penems to other antibiotics. Focused surveillance and monitoring efforts will aid in optimizing the clinical use of penems [13].

The World Health Organization (WHO) priority list of pathogens serves as a driving force and guideline for developing new antimicrobials and combining new and existing agents to address the rising threat of MDR Gram-negative pathogens [55]. Also, the WHO’s annual pipeline report (2021) highlights the inadequate development of new antibacterial agents to address the growing threat of antibiotic resistance. The report characterizes the antibacterial clinical and preclinical pipeline as stagnant and insufficient to address global demands. It emphasizes the small number of approved antibiotics, most of which belong to existing classes with established mechanisms of antimicrobial resistance [90]. Recently, Brüssow H. (2024), in his review analysis, affirmed that the research efforts were primarily carried out by academic researchers and biotech companies with constrained financial resources. Consequently, the stagnation in new antibiotic development, often referred to as the drying up of the pipeline, appears to be more due to the insufficient mobilization of the necessary monetary resources to bring the discoveries to market rather than a lack of scientific understanding, despite recent financial initiatives from the public sector [91].

## 5. Materials and Methods

The review is based on collected references from various databases, including Clarivate Analytics, ScienceDirect, PubMed, and Google Books. The primary keywords used were “penem”, “carbapenem”, “β-lactam antibiotics“, “sulopenem“, “etzadroxil“, “probenecid”, “faropenem“, “urinary tract infections“, and “UTIs“, combined with specific terms regarding new molecules in development. Additionally, other relevant keywords such as “drug candidates”, “drug discovery”, “drug design”, “antibacterials”, and “antibacterial resistance” were considered. Among the obtained references, those concerning penem derivatives that proved antibacterial activity were selected. Chemical structures were drawn using Biovia Draw 2024 (https://discover.3ds.com/biovia-draw-academic-thank-you accessed on 18 February 2025) [69]. The stereochemistry of the compounds and some calculations were checked with MarvinSketch 23.10 (https://chemaxon.com/marvin accessed on 28 February 2025) [48]. The IUPAC names of some compounds were obtained from the PubChem database (https://pubchem.ncbi.nlm.nih.gov/ accessed on 29 January 2025) [5].

## 6. Conclusions

Penems’ structural similarity to penicillins lies in their fused thiazoline, but with a double bond, making them more chemically reactive. Unlike naturally occurring carbapenem antibiotics, penems are synthetic compounds. The structure features share a bivalent isostere structure, where a sulfur atom replaces a methylene group in the five-membered ring fused to the β-lactam ring. The modification introduces specific reactivity and resistance characteristics, such as resistance to bacterial or mammalian β-lactamases.

Faropenem, the first penem antibiotic approved in Japan, is efficient against many Gram-positive and Gram-negative bacteria, including those β-lactamase-producing ones. Faropenem was approved to treat UTIs, respiratory tract infections, skin and skin structure infections, and gynecological infections in Japan, India, and China. Structurally, faropenem is characterized by a tetrahydrofuran ring at the C3 position, contributing to its chemical stability and resistance to kidney DHP-1 hydrolysis. The tetrahydrofuran ring at the C3 position makes it more stable than carbapenems and less likely to cause CNS effects. However, its prodrug derivative, faropenem medoxomil, was rejected by the FDA in 2006.

More recently, in 2024, the FDA approved a combination of sulopenem etzadroxil and probenecid (Orlynvah) for oral use, this being the first oral penem authorized for use in the USA. Combining sulopenem etzadroxil and probenecid improves sulopenem absorption and increases plasma concentrations. Sulopenem’s antibacterial activity covers a broad spectrum of Gram-positive and Gram-negative bacteria, including ESBL-producing *Enterobacterales* and fluoroquinolone-resistant strains. However, it is ineffective against *Pseudomonas*, *Enterococcus*, MRSA, and carbapenem-resistant *Enterobacteriaceae*. Orlynvah was approved for treating uncomplicated UTIs caused by *Escherichia coli*, *Klebsiella pneumoniae*, or *Proteus mirabilis* for adult women with minimal or no other options for oral antibiotic treatment.

Over time, other penem derivatives have been studied to obtain effective antibiotics. The essential structural characteristics responsible for antibacterial activity resulting from all the penem compounds discussed in our review are summarized further.

MEN 10700 exhibits broad-spectrum antibacterial activity, being effective against Gram-positive and Gram-negative bacteria; MEN 11505 is a compound optimized for better bioavailability as an oral prodrug of MEN 10700. A 2-benzyl imidazole derivative demonstrated potent β-lactamase inhibitory activity, broadening the spectrum of activity. Tricyclic substitutions led to 5,5,6-fused tricyclic-6-methylidene penems with enhanced β-lactamase inhibitory activity, especially against class A and C enzymes. The changes at the C6 position improved binding affinity to bacterial signal peptidase and enhanced antibacterial activity. A bicyclic-6-methylidene structure (BLI-489) inhibits class A, C, and D β-lactamase enzymes, showing potential in combination therapies. A ferrocenyl group at the C3 position offers superior antibacterial efficacy and stability against DHP-1 compared to faropenem. The bicycle-substituted 6-methylidene-penems obtained by pyrazolo [1,5-*c*][1,3]thiazine substitution efficiently inhibit the BlaC enzyme, suggesting the significant growth inhibition of *Mycobacterium tuberculosis*. The study of T405 and derivatives shows that azetidine and 2-thiazoline ring modifications maintain or improve antimicrobial activity. The five-membered rings (e.g., T425 and T426) exhibit intense activity against *Mycobacterium tuberculosis* and *Mycobacteroides abscessus*. Non-aromatic modifications generally show better antimicrobial results. The compounds with lower PPB demonstrate better activity in the presence of serum, indicating potential in vivo efficacy.

Concerning the future of penem antibiotics, we highlighted their promising role in combating bacterial resistance. The paper underlines the importance of ongoing research into and development of penem derivatives to enhance their efficacy, oral bioavailability, and antibacterial coverage. Additionally, some penem derivatives show promise as potent β-lactamase inhibitors, which could further extend their clinical utility. Another promising strategy involves combination therapies, pairing novel penem derivatives with β-lactamase inhibitors to combat resistant bacterial strains more effectively. Continued investment in research is essential to optimize the clinical use of penems and address the growing threat of antibiotic resistance.

## Figures and Tables

**Table 1:** Essential structural elements of penems and carbapenems used in therapy (approved by the FDA, EMA or in the countries of origin) [5,18,19,20,21,22].

		Positions						
Class	Compounds	C2	C2–C3	C3	C4 or S4	C5	C6	C7
Penems (Thiopenems)	Faropenem	Carboxyl	Double bond	[(2*R*)-oxolan-2-yl]	-S- (thia)	H (*R*)	[(1*R*)-1-hydroxyethyl]; H (*S*)	Oxo
	Sulopenem	Carboxyl	Double bond	[(1*R*,3*S*)-1-oxothiolan-3-yl]sulfanyl	-S- (thia)	H (*R*)	[(1*R*)-1-hydroxyethyl]; H (*S*)	Oxo
Carbapenems	Biapenem	Carboxyl	Double bond	(6,7-dihydro-5*H*-pyrazolo [1,2-*a*][1,2,4]triazol-4-ium-6-ylsulfanyl)	-CH(methyl)- (4*R*)	H (*R*)	[(1*R*)-1-hydroxyethyl]; H (*S*)	Oxo
	Doripenem ^1^	Carboxyl	Double bond	[(3*S*,5*S*)-5-[(sulfamoylamino)methyl]pyrrolidin-3-yl]sulfanyl	-CH(methyl)- (4*R*)	H (*R*)	[(1*R*)-1-hydroxyethyl]; H (*S*)	Oxo
	Ertapenem	Carboxyl	Double bond	[(3*S*,5*S*)-5-[(3-carboxyphenyl)carbamoyl]pyrrolidin-3-yl]sulfanyl	-CH(methyl)- (4*R*)	H (*R*)	[(1*R*)-1-hydroxyethyl]; H (*S*)	Oxo
	Imipenem	Carboxyl	Double bond	[2-(aminomethylideneamino)ethylsulfanyl]	-CH_2_-	H (*R*)	[(1*R*)-1-hydroxyethyl]; H (*S*)	Oxo
	Meropenem	Carboxyl	Double bond	[(3*S*,5*S*)-5-(dimethylcarbamoyl)pyrrolidin-3-yl]sulfanyl	-CH(methyl)- (4*R*)	H (*R*)	[(1*R*)-1-hydroxyethyl]; H (*S*)	Oxo
	Panipenem	Carboxyl	Double bond	[(3*S*)-1-ethanimidoylpyrrolidin-3-yl]sulfanyl	-CH_2_-	H (*R*)	[(1*R*)-1-hydroxyethyl]; H (*S*)	Oxo

^1^ Withdrawn in EU.

**Table 2:** Physicochemical properties of sulopenem (CAS Number: 120788-07-0); Ref. = references.

No.	Properties	xx	Ref
1	Molecular weight	349.5 g/mol	[5]
2	Water solubility	9.29 mg/mL (ALOGPS)	[5]
3	DMSO solubility	2 mg/mL	[47]
4	p*K*_a_ (Strongest Acidic)	3.65 (Chemaxon)	[5]
5	p*K*_a_ (Strongest Basic)	−2.8 (Chemaxon)	[5]
6	p*I* (isoelectric point)	1.65	[48]
	log*P*	−0.21 (ALOGPS); −1.3 (Chemaxon)	[49]
	log*S*	−1.6 (ALOGPS)	[49]
7	log*D* (pH 7.4)	−4.81	[48]
8	Boiling point	693.1 ± 55.0 °C (predicted)	[50]
9	Density	1.74 ± 0.1 g/cm^3^ (Predicted)	[50]
10	Form	Solid/powder	[47,50]
11	Color	White to yellow/white to beige	[47,50]
12	Storage temperature	Store at −20 °C; after the delivery date, it can be stored for up to six months.	[47,50,51]
13	Solutions	After being made, the stock solutions are used within a month after being aliquoted and kept at −20 °C or below in firmly sealed vials; solutions should be prepared and used on the same day wherever possible.	[51]

**Table 3:** Pharmacokinetics of intravenous sulopenem in healthy subjects [19].

No.	Pharmacokinetic Parameters	Values
1	Volumes of distribution (Vd)	15.8–27.6 L
2	Total drug clearances (CLT)	18.9–24.9 L/h
3	Protein binding	~10%
4	Elimination half-lives (t½)	0.88–1.03 h
5	Estimated renal clearance (CLR)	8.0–10.6 L/h
6	Dose recovered unchanged in the urine	35.5% ± 6.7% of a 1000 mg

**Table 4:** Comparison of penems and carbapenems antibiotics [9,13,16,18,20,21,33,40,42].

Feature	Penems	Carbapenems
Stability to DHP-1	Generally stable (e.g., faropenem, sulopenem)	Some require co-administration with DHP-1 inhibitors (e.g., imipenem + cilastatin)
Oral bioavailability	Oral forms (e.g., sulopenem etzadroxil, faropenem medoxomil)	Mostly IV ^1^; poor oral bioavailability
β-Lactamase resistance	Moderate to high (depends on side chains)	High, especially against ESBLs and AmpC
Spectrum of activity	Broad: Gram-positive, Gram-negative, anaerobes; some MDR strains	Very broad: Gram-positive, Gram-negative, anaerobes, MDR strains
Representatives	Faropenem, Sulopenem	Biapenem, Doripenem, Ertapenem, Imipenem, Meropenem, Panipenem
FDA Approved representatives	Sulopenem	Doripenem, Ertapenem, Imipenem, Meropenem
Resistance mechanisms	Altered PBPs, carbapenemase production, efflux pumps, reduced outer membrane permeability, porin loss or modification; combined mechanisms	Altered PBPs, carbapenemase production, efflux pumps, porin loss or modification; combined mechanisms
Clinical use	UTIs, respiratory infections, skin and soft tissue infections, gynaecological infections	Broad use: sepsis, respiratory tract infections, intra-abdominal infections, meningitis, skin and soft tissue infections, complicated UTIs
Side effects	Similar to β-lactam antibiotics; sulopenem: diarrhoea	Similar to β-lactam antibiotics; imipenem may cause seizures in high doses
Combination therapies	Sulopenem with probenecid (organic anion transport inhibitor that delays renal excretion)	Some with β-lactamase inhibitors (e.g., relebactam); some with DHP-1 inhibitors (e.g., imipenem + cilastatin)

^1^ IV—intravenous.

## Abbreviations

The following abbreviations are used in this manuscript:

CDSCO	Central Drugs Standard Control Organization
CNS	Central Nervous System
DHP-1	Dehydropeptidase-I
EMA	European Medicines Agency
ESBL	Extended-Spectrum Beta-Lactamase
EU	European Union
FDA	Food and Drug Administration
KES	Klebsiella, Enterobacter, and Serratia
MDR	Multidrug-Resistant
MIC	Minimum Inhibitory Concentration
MRSA	Methicillin-resistant *Staphylococcus aureus*
MSSA	Methicillin-Sensitive *Staphylococcus aureus*
Mtb	*Mycobacterium tuberculosis*
OAT	Organic Anion Transporter
PBPs	Penicillin-Binding Proteins
PPB	Plasma Protein Binding
UTIs	Urinary Tract Infections
